# Fast, Automated, Knowledge-Based Treatment Planning for Selecting Patients for Proton Therapy Based on Normal Tissue Complication Probabilities

**DOI:** 10.1016/j.adro.2022.100903

**Published:** 2022-01-28

**Authors:** Roni Hytönen, Marije R. Vergeer, Reynald Vanderstraeten, Timo K. Koponen, Christel Smith, Wilko F.A.R. Verbakel

**Affiliations:** a Varian Medical Systems Finland, Helsinki, Finland; b Department of Radiation Oncology, VU University Medical Center, Amsterdam, The Netherlands; c Varian Medical Systems Belgium, Diegem, Belgium; d Varian Medical Systems, Palo Alto, CA, USA

## Abstract

**Purpose:**

Selecting patients who will benefit from proton therapy is laborious and subjective. We demonstrate a novel automated solution for creating high-quality knowledge-based plans (KBPs) using proton and photon beams to identify patients for proton treatment based on their normal tissue complication probabilities (NTCP).

**Methods and Materials:**

Two previously validated RapidPlan PT models for locally advanced head and neck cancer were used in combination with scripting to automatically create proton and photon KBPs for 72 patients with recent oropharynx cancer. NTCPs were calculated for each patient based on the KBPs, and patient selection was simulated according to the current Dutch national protocol.

**Results:**

The photon/proton KBP exhibited good correlation between predicted and achieved organ-at-risk mean doses, with a ≤5 Gy difference in 208/196 out of 215 structures relevant for the head and neck cancer NTCP model. The proton KBPs yielded on average 7.1/6.1/7.6 Gy lower dose to salivary/swallowing structures/oral cavity than the photon KBPs. This reduced average grade 2/3 dysphagia and xerostomia by 7.1/3.3 and 5.5/2.0 percentage points, resulting in 16 of 72 patients (22%) being indicated for proton treatment. The entire automated process took <30 minutes per patient.

**Conclusions:**

Automated support for decision making using KBP is feasible and fast. The planning solution has potential to speed up the planning and patient-selection process significantly without major compromises to the plan quality.

## Introduction

Recent increases in proton treatment capacity and advancement in radiation therapy treatment modalities have made it possible to treat more patients with proton therapy.^1^ However, some patients may not receive additional or sufficient benefit from proton over conventional treatment regarding probability of tumor control or risk of toxicity. This, together with the higher cost of treatment and limited capacity of the proton centers, necessitates patient selection.^2^, ^3^, ^4^

One objective way to select the patients who can benefit most from proton therapy is by using a model-based indication methodology. The methodology could be based on the estimated reduction in normal-tissue complication probability (NTCP).^2^, ^3^, ^4^, ^5^, ^6^ This selection process necessitates creation of high-quality photon and proton treatment plans for individual patients, which can be laborious, time consuming, and prone to bias. Because the plan quality may vary among planners, institutions, and experience per modality, comparison of proton and photon modalities can become unreliable.^7^, ^8^, ^9^

One solution to reduce the plan quality variations is to automate or semiautomate the planning process, for example by using a knowledge-based planning solution. Previous studies have shown such solutions to significantly speed up the planning process and to yield high-quality photon and proton treatment plans, provided that the library of plans they're based on is of sufficiently high quality.^10^, ^11^, ^12^, ^13^, ^14^, ^15^

In this proof-of-principle study, we investigated if combining a commercially available knowledge-based planning solution with task automation tools allows creation of very efficient semi- or fully automated workflows for locally advanced head and neck cancer (HNC). This can aid and drastically speed up the decision-making process between photons and protons, and base it on quantitative data. We have further investigated which proportion of patients with HNC is eligible for proton therapy according to the current Dutch guidelines^5^ based on a large, representative group of patients.

## Methods and Materials

**Fig. 1:**
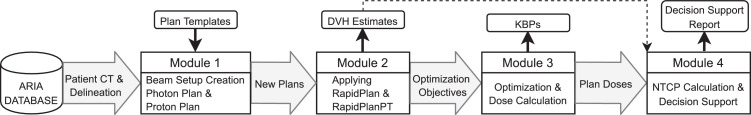
Visualization of the decision support system pipeline. *Abbreviation:* NTCP = normal tissue complication probability.

We designed a modular automated knowledge-based treatment planning solution (Fig 1) to support the patient selection between protons and photons. The system was implemented using Eclipse Scripting Application Programming Interface (Varian Medical Systems, Palo Alto, CA),^16^^,^^17^ and it is designed to use a fully delineated patient computed tomography scan and externally defined plan templates describing the desired treatment plans as a starting point. The knowledge-based planning parts of the system are RapidPlan (Varian Medical Systems) and RapidPlan for protons (RapidPlan PT). Both RapidPlan systems use libraries of previously created treatment plans to construct predictive models for each organ at risk (OAR) based on the dosimetry and geometry of the plans, taking into account the physical properties of the radiation. These models are then used to predict a range of possible achievable dose-volume histograms (DVHs) for the OARs for a specific patient, based on their geometry. The optimization objectives are subsequently based on the lower DVH prediction range.^10^^,^^14^

The pipeline consists of 4 interlinked modules. Module 1 imports the patient computed tomography and delineations from a database, reads the plan templates, and uses these to generate the beam setup for photon and proton treatment plans. Modules 2 and 3 together apply RapidPlan and RapidPlan PT for OAR DVH prediction, set the optimization objectives, optimize the plan, and perform the final dose calculation to generate knowledge-based plans (KBPs). The final module reads the proton and photon KBP doses, computes the NTCP values, and indicates the patient for the preferred treatment modality according to the Dutch standard. The modular design also allows for the RapidPlan-generated DVH estimates to be used for computing the NTCP estimates.

### Treatment plans

The plan templates were configured to generate treatment plans in line with the clinical practice at the VU Medical Center for patients with locally advanced HNC; a 3-field intensity modulated proton therapy (IMPT) plan with beams at ±45° and 180° and a volumetric modulated arc therapy (VMAT) plan using 2 full arcs and 6 MV photons. The IMPT plans were created using pencil beam spot scanning from the ProBeam proton system (Varian Medical Systems). Each IMPT field included a proximal/distal/lateral target margin of 2/3/5 mm. The plans used simultaneous integrated boost technique, delivering 70/54.25Gy to the boost/elective planning target volumes (PTV_B_/PTV_E_) in 35 fractions. PTV_B_ consisted of the gross tumor volume, expanded with a 5-mm margin to clinical target volume (CTV) (edited for anatomic boundaries) and a 4- to 5-mm PTV margin. PTV_E_ consisted of the gross tumor volume expanded with a 5- to 10-mm margin to CTV plus elective nodal CTV, both plus a 4- to 5-mm PTV margin, minus PTV_B_. An additional 5-mm wide ring (PTV_O_) around PTV_B_ was subtracted from PTV_E_ to allow for a steep dose fall-off in the planning process. Finally, the PTVs were cropped to 5 mm within the body surface, and their union (PTV_COMP_) was used as the field target for all fields. Planning aim was to cover ≥98% of boost and elective volumes with ≥95% of the dose (V95% ≥ 98%). Dose objectives for OAR were maximum dose of 50 Gy to spinal cord expanded with a 3-mm margin and 54 Gy to brain stem expanded with a 3-mm margin but preferably lower; and as low as possible mean dose to both parotid glands, both submandibular glands, oral cavity (OC), and individually contoured swallowing muscles.^18^

RapidPlan models were trained on 112 clinical HNC 2-arc RapidArc photon and 50 manually planned 3-field IMPT proton plans, respectively. Both models are based on libraries of larynx, pharynx, hypopharynx, and oropharynx cancer plans, and were previously proven to be of high quality.^14^^,^^15^ The models aimed to spare the mean dose to all OARs without specific aim to reduce the NTCP, and they focused on sparing the salivary glands, while the swallowing structures had slightly lower priority.^19^ The photon library plans were made using an in-house developed automated interactive optimization^20^ and the resulting plan quality of the model (with more or fewer library plans) was validated earlier.^21^^,^^22^ The photon model was based on plans made only briefly after introduction of OC sparing, when the OC had a lower priority than the salivary glands. The contemporary clinical photon treatment plans aim to spare OC more aggressively than what was done with the plans in the photon model library. We therefore reduced the predicted OC mean dose by 5 Gy and used a 20% higher priority than in the earlier models. As a comparison, plans were also made using the original photon model.

The proton KBP optimization was performed using the Varian Eclipse nonlinear universal proton optimizer 16.0.2 with nonrobust multifield optimization, followed by dose calculation using proton convolution superposition algorithm 16.0.2. Possible range uncertainties were not taken into account and plans were optimized by defining dose aims for the same PTV structures as used for the photon plans. The photon KBPs were optimized using photon optimizer 16.0.2, and the dose was calculated with Acuros XB 16.0.2. Furthermore, a subsequent “continue optimization” with increased photon PTV objective priorities was performed to improve the PTV dose homogeneity and coverage. The plans were normalized to PTV_B_ V_95%_ = 98%. Examples of the proton beam arrangement and dose distributions for both proton and photon KBPs are provided in Supplementary Material A, Figures A1–A3.

### NTCP models and treatment modality indication

**Fig. 2:**
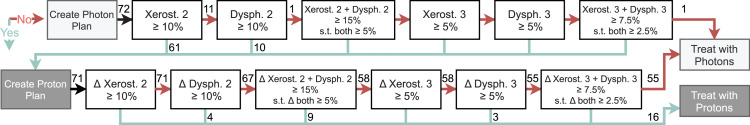
Decision process for selecting patients for photon/proton treatment and the number of patients indicated at each decision node.^5^ Delta indicates the difference between the NTCPs of the 2 plans (proton plan NTCP minus photon plan NTCP). *Abbreviation:* NTCP = normal tissue complication probability.

For the auto-generated KBPs, NTCP for grade 2 and 3 xerostomia and swallowing dysfunction at 6 months after the treatment were calculated using the models adopted by the Dutch radiation oncology society.^5^ The results were compared against the treatment modality selection flowchart (Fig 2).

NTCP values for each endpoint were evaluated for each patient as $NTCP=\frac{1}{1\;+e^{-S}}$ with linear predictor (S) defined as $S=\beta_{0}+\Sigma_{i}\left(\beta_{i}\cdot x_{i}\right),$ where $\beta_{0}$ is a model constant, $\beta_{i}$ are variable-wise coefficients, and $x_{i}$ are the variables that may be continuous (dose metrics) or categorical (patient demographic data).^5^ The OAR dose metrics involved in the different NTCPs are the mean doses to both parotid glands, submandibular glands, OC, and pharyngeal constrictor muscles. Details of the model parameters and coefficient values are presented in Supplementary Material B, Table B1. According to the Dutch model, proton treatment is indicated if there is at least 10%/5% lower NTCP for grade 2/3 complications in the proton plans, or if the sum of both grade 2/3 NTCPs is 15%/7.5% lower for proton plans.

We investigated which fraction of patients would be eligible for proton therapy using the original RapidPlan models and the model with the adapted OC objectives. In addition, we also studied whether only the RapidPlan-generated DVH estimates can be used for evaluating the NTCP values by comparing the calculated KBP results to ones acquired by using the OAR mean doses predicted by RapidPlan. The middle of the DVH-prediction range was used to represent the predicted mean dose. Although on average the achieved mean doses were similar to the predicted ones, their difference could be dose dependent. For that reason, a dose-dependent correction factor was calculated from the difference between predicted and achieved OAR mean doses, and it was applied to both photon and proton DVH predictions. The treatment modality selection was simulated using the predictions for both plans and with predictions for protons and KBP for photons.

### Evaluation

A group of 72 subsequent patients with locally advanced oropharynx cancer, not included in either of the RapidPlan models, was chosen arbitrarily for the evaluation. All patients signed an informed consent before starting therapy that their data could be used within the department's research program. The medical ethics committee of Amsterdam University Medical Centers exempted this work from requiring their official approval. The patients had been treated with 2-arc VMAT between 2016 and 2019 using the same fractionation scheme as for the model patients.

Photon and proton KBPs and an NTCP comparison were created for each patient of the evaluation group, and none of the KBPs were adjusted manually. For the NTCP computations, all patients were assumed to be free of any pre-existing conditions affecting the model baseline values.

A small number of patient OAR delineations did not include all structures required for the calculation of NTCP values, often when the respective OAR was completely within the PTV. The contributions of those structures to the respective NTCP values were omitted from the comparisons, although we later verified if the photon-proton selection would change if we assumed they had received the average PTV dose.

## Results

The time to automatically create both plans and perform the selection was <30 minutes per patient.

**Fig. 3:**
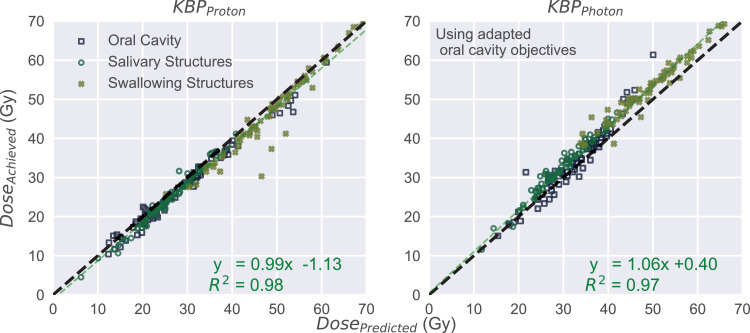
Achieved versus predicted organ-at-risk mean doses for all patients. Identity line shown (black line) along with linear fit (green line). *Abbreviation:* KBP = knowledge-based plan.

**Fig. 4:**
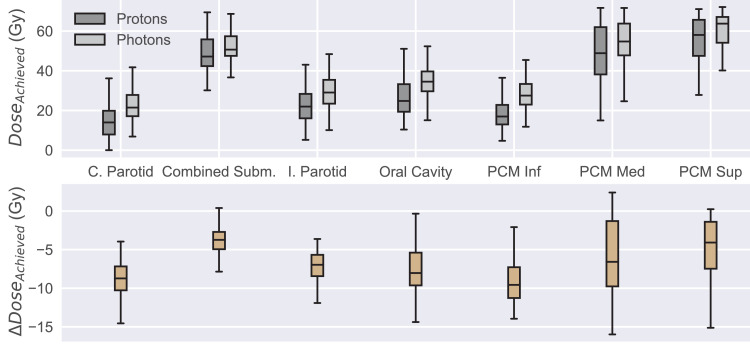
Box-whisker plots for all 72 patients indicating the achieved proton and photon plan organ-at-risk mean doses (top) and their differences (bottom, proton plan dose minus photon plan dose). Dark line in the middle of the box indicates the median, the top/bottom of the box indicates 75th/25th percentile, and the whiskers indicate the range of the data. *Abbreviation:* PCM =Pharyngeal Constrictor Muscle.

Model-based DVH estimates for both proton and photon KBPs showed good correlation (R^2^ of 0.98 and 0.97, respectively) between the predicted and achieved OAR mean doses (Fig 3), with protons predicting slightly higher and photons slightly lower mean doses than what was eventually achieved. Altogether, the differences in proton/photon predicted and achieved OAR mean doses were ≤5 Gy for 72/67 out of 72 combined salivary structures, 65/62 out of 71 combined swallowing structures, and 71/67 out of 72 OC structures. The proton KBPs showed overall lower achieved OAR mean doses (Fig 4), with an average of 7.1/6.1/7.6 Gy reduction in salivary/swallowing structures/OC. The salivary/swallowing structures/OC had substantial (≥5 Gy) mean dose differences in favor of protons in 64/42/56 out of 72/71/72 cases.

**Fig. 5:**
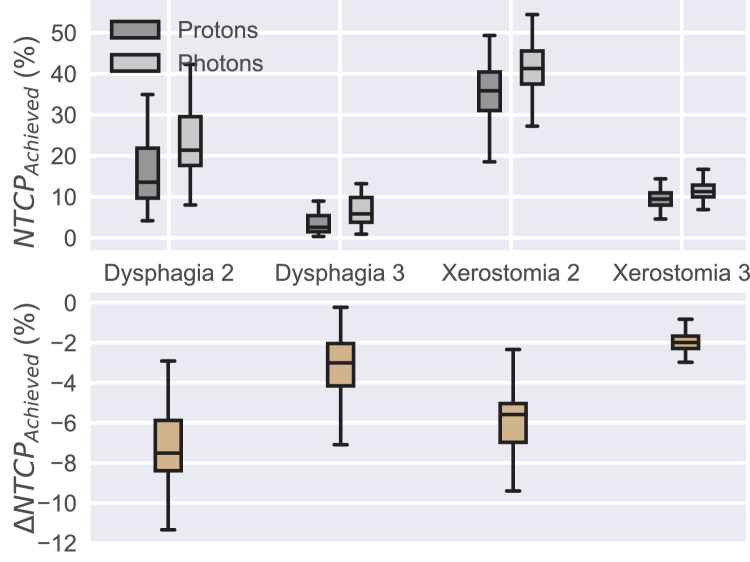
Achieved proton and photon plan NTCP values (top) and their difference (proton plan NTCP minus photon plan NTCP) (bottom). *Abbreviation:* NTCP = normal tissue complication probability.

The proton/photon NTCP values (Fig 5) mirrored the OAR mean dose differences, with the average grade 2 dysphagia and xerostomia being 15.9/23.0 and 35.6/41.5 percentage points, respectively. The average grade 3 dysphagia and xerostomia were 4.7/8.0 and 9.5/11.5 percentage points, respectively.

The NTCP values indicated 16 out of the 72 patients for proton therapy (Fig 2), following the Dutch guidelines. Our pipeline deviates from the suggested process by first creating both plans and then using the photon NTCPs to see whether the comparison is warranted. For this cohort, all but 1 patient were indicated for the NTCP comparison part of the decision-making workflow. The patients were finally indicated for proton therapy owing to reduced probability of both grade 2 and 3 dysphagia (4 and 3 patients, respectively) and owing to the reduced combined probability of grade 2 dysphagia and xerostomia (9 patients). The selection results did not change depending on how the missing OARs were handled.

If patient selection was based on the original RapidPlan PT model, without stronger OC sparing, 40 out of 72 patients would have qualified for proton therapy. The extra OC objective reduced the OC mean dose by 5.0 Gy, while the dose to parotid glands increased by 0.8 Gy on average.

**Fig. 6:**
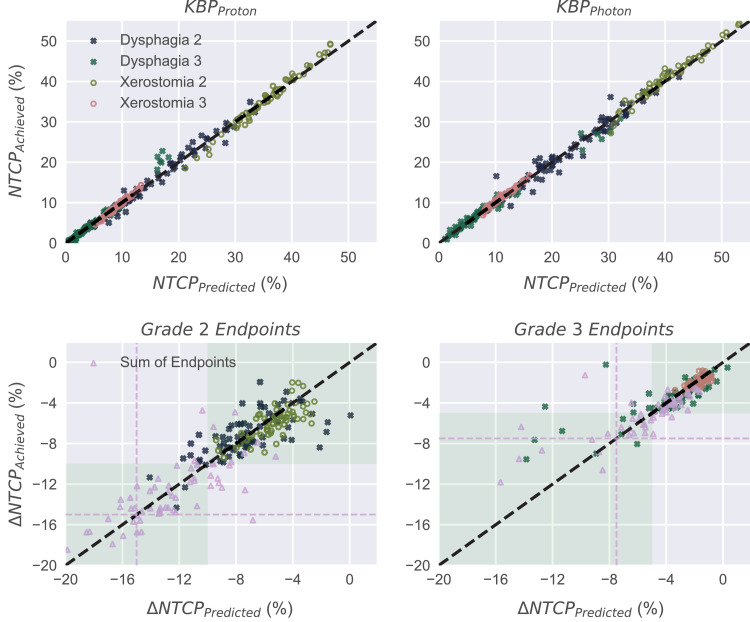
Achieved versus predicted NTCP values for all patients (top) and achieved versus predicted absolute differences in the patient-wise NTCP values (proton plan minus photon plan) (bottom). The decision threshold levels of ∆NTCP are marked in green for xerostomia and dysphagia. The triangles and dashed horizontal/vertical lines in the lower images indicate the sum of the 2 endpoints and the respective decision thresholds. *Abbreviations:* KBP = knowledge-based plan; NTCP = normal tissue complication probability.

The proton/photon prediction ranges for the grade 2 and 3 endpoints have variances of 1.5/2.2 and 0.85/0.59 percentage points, respectively (Fig 6). For ΔNTCP, the variances accumulate, yielding 2.9/2.1 percentage points for grade 2/3 dysphagia and xerostomia and 5.1/4.2 percentage points for the sum of the 2. Based on the NTCP predictions, 13/9 and 47/3 patients were indicated correctly/incorrectly to proton and photon treatment, respectively. If the DVH estimates were used only for the proton plan while the actual DVHs from KBPs were used for the photon plan, the number of patients indicated erroneously for proton therapy dropped to 6.

## Discussion

In this proof-of-principle study, we demonstrated that commercially available scripting and knowledge-based planning tools can be used to create a fast, fully automated decision support pipeline for the selection between photon and proton treatments for HNC. Whereas conventional treatment modality comparisons require burdensome creation of patient-specific plans, the suggested pipeline can produce them autonomously, which has potential to drastically improve the planning efficiency and reduce variations. As the quality of the produced treatment plans depends heavily on the quality of the model library plans and the beam arrangement, manual review and occasional adjustments of the KBPs remains necessary.

We have demonstrated the feasibility of RapidPlan for decision making between protons and photons, but at that time we only had access to a photon RapidPlan algorithm.^23^ Now, the photon and proton plans have been made using their proper RapidPlan algorithms, and for a large, representable group of patients. The OAR mean-dose predictions showed a good degree of correlation with the achieved KBP dose distributions for both photon and proton plans. The dose prediction-based patient selection managed to indicate 13 out of the 16 KBP-indicated patients (81%) for proton therapy. The accuracy of the predictions was, however, insufficient to be solely used for the patient selection process, as even after applying the correction factor, 9 and 3 patients were falsely indicated to proton and photon treatment, respectively. A better result was achieved if the RapidPlan-estimates were used only for the proton plan, leading to 6 patients being falsely indicated to protons, as using estimates only for 1 of the 2 plans drastically reduces the accumulation of errors in the ΔNTCP. The error accumulation is a major hindrance in using the RapidPlan predictions for the patient selection, but we found minor benefit in using RapidPlan prediction range instead of the predicted mean dose (Supplementary Material C).

To place this research in context, the first experience with model-based selection of patients with HNC for proton therapy in the Netherlands was recently reported,^6^ concluding the model-based selection to be clinically feasible, albeit resource intensive. They found a larger portion of patients (35% as opposed to our 22%) to qualify for protons, and both studies found dysphagia-related toxicities to be the more common reason for patients being indicated for protons. The results from their study are, however, not directly comparable with ours due to numerous differences in methodology, including them using the Dutch NTCP model from year 2017, proton plans with 6 or more beams, and the actual baseline conditions of the patients, while we used the 2019 version of the NTCP model, 3-field proton plans, and assumed all patients to be free of baseline conditions. Furthermore, our research illustrates that when the OC was spared less intensively, the number of patients selected for protons increased to 56%, demonstrating the necessity to aim for optimal OAR sparing in the photon plans.

Kouwenberg et al^24^ have recently demonstrated how their in-house automatic multicriterial optimizer together with Bayes classification on preselecting patients for plan comparison reduced the number of unnecessary manually planned IMPTs. Although our auto-generated KBPs are intended to offer the user clinically relevant treatment plans with both modalities to aid the planner in about 30 minutes, it would be of interest to see how the auto-generated IMPTs perform in a similar preselection study, as the entire IMPT-portion of the pipeline takes about 10 minutes. Also, others have suggested different approaches for planning automation and decision support,^25^, ^26^, ^27^ but to our knowledge we are the first to combine plan creation, knowledge-based planning, and decision support in a single pipeline.

The mean NTCP values achieved with the OC-sparing photon plans in this study were 1% to 10% lower than their reported prevalence.^5^ The lower prevalence of xerostomia is partially explained by the reduced dose to OC, but the majority of differences are most probably due to the favorable baseline assumptions, as attributing the cohort with baseline dysphagia or xerostomia can increase the respective NTCP by at least 1% to 10%, depending on what portion of the patients the conditions are assigned to. We have contoured the OC not according to Brouwer et al,^18^ which should be used in the NTCP, but slightly larger, including the buccal mucosa and part of the lips. The OC dose according to Brouwer would therefore be a bit higher for both the proton and photon plans, and could lead to more patients being indicated for protons.

The major limitations of this study were the lack of robust optimization, lack of externally validated RapidPlan models, a proton model that is based on 3 fixed beam directions, and the models not being optimized for the lowest NTCP. Furthermore, the proton beam angles were not adapted in case of dental amalgam presence. The robustness of the model for gantry angle changes >30° has not yet been evaluated. On the other hand, robust optimization of photon plans and planning with more than 2 arcs may also allow for lower OAR doses in the VMAT plans. The combinations make it hard to predict what could happen to the NTCP comparison. The plans created as a part of this study were not robustly optimized, but preliminary tests have shown that RapidPlan PT models based on nonrobustly optimized plans can be used for robust optimization,^15^ and robust optimization and assessment of robustness criteria would be valuable additions to the automated pipeline.^28^ The RapidPlan PT model has been compared in 3 renowned proton centers,^15^ but it has not been compared with proton plans from centers in the Netherlands, nor has the photon RapidPlan model been externally validated. In addition, instead of being optimized for the lowest NTCP, both models are based on mean dose reduction of a large quantity of individual OARs; using NTCP-based optimization could change the priorities between certain OARs and lead to reduced NTCP.

Regarding the clinical applicability of the approach, the automated planning process, as well as the created photon and proton KBPs, were representative of the current clinical approach for HNC planning, and in practice we have found the need for manual adapting of the KBPs rare. While the 3-field proton KBPs used here may be suboptimal, as discussed previously, previous studies have found minor to no benefit in OAR sparing by using 5 or 9 fields instead of 3.^29^^,^^30^ Additionally, in a previous study, the KBPs were found to compare equally or outperform manual plans, although for a few patients the manual plans outperformed the KBP slightly.^15^

The applicability of the NTCP comparison is limited in cases where not all OARs required for the calculation of NTCP values are delineated, which may be the case, for example, when the OAR is within PTV. In the 72-patient cohort used in this study, ipsilateral/both submandibular gland contours were missing for 6/5 patients and pharyngeal constrictor muscle contours for 1 patient. For this cohort, we found there to be no difference in photon-proton selection results depending on whether the missing OARs were ignored or assigned average PTV dose.

The presented pipeline is versatile and can be adapted to other treatment sites. Each module can also be run individually, allowing for manual review and adjustments, if needed. The level of automation could be further increased, for example, by adding a deep-learning segmentation solution to it.^31^ Additionally, the pipeline could greatly benefit from beam angle optimization, field-specific targets, and geometric outlier detection. Although different approaches for beam angle optimization have been suggested,^32^^,^^33^ the applicability of KBP in cases where beam angles differ from the ones used in the model library is not yet well understood and would be of interest for future research. Depending on the shape and location of the PTV, certain cases would benefit from using field-specific targets instead of composite-PTV. Likewise, automatically detecting and removing the geometric outliers could increase the DVH estimate and optimization objective quality.

Although we used analytical algorithms for optimization and dose calculation in this study, the pipeline is algorithm agnostic, and, for example, the used dose calculation algorithm could be replaced by a Monte Carlo (MC). MC dose calculations, however, do not typically change the shape of the dose distribution, but the DVH can be slightly different from a DVH based on another algorithm.^34^ Therefore, the most optimal would be to have a plan library for which the dose calculation was done using MC, and an MC dose calculation in the optimizer. In case MC was used only for the final dose calculation but not in the optimizer, there would most likely be a discrepancy between the optimizer dose at end optimization and final dose calculation.

## Conclusion

The degree of automation provided by scripting and knowledge-based planning presents a grand opportunity to automate significant portions of the treatment planning process. This can aid the work of more established radiation therapy institutions, as well as bring in-house decision support within the grasp of less experienced facilities. The knowledge-based predictions could be selectively used to study the need for creating comparative proton plans and the robustness of the prediction-based modality selections, and they can help to better select the patients who would benefit from proton therapy.

## Appendix Supplementary materials

Image, application 1

Image, application 2

Image, application 3

## Footnotes

Supplementary material associated with this article can be found in the online version at doi:10.1016/j.adro.2022.100903.
