# Supplementary material for: Fast, Automated, Knowledge-Based Treatment Planning for Selecting Patients for Proton Therapy Based on Normal Tissue Complication Probabilities
Source: Adv Radiat Oncol. 2022 Jan 28;7(4):100903. doi: 10.1016/j.adro.2022.100903 (PMC8904224; doi:10.1016/j.adro.2022.100903)
Supplement: Supplementary file 1 [file mmc1.pdf]

Supplementary Material A: Example auto-generated knowledge-based plans for a patient indicated for proton therapy

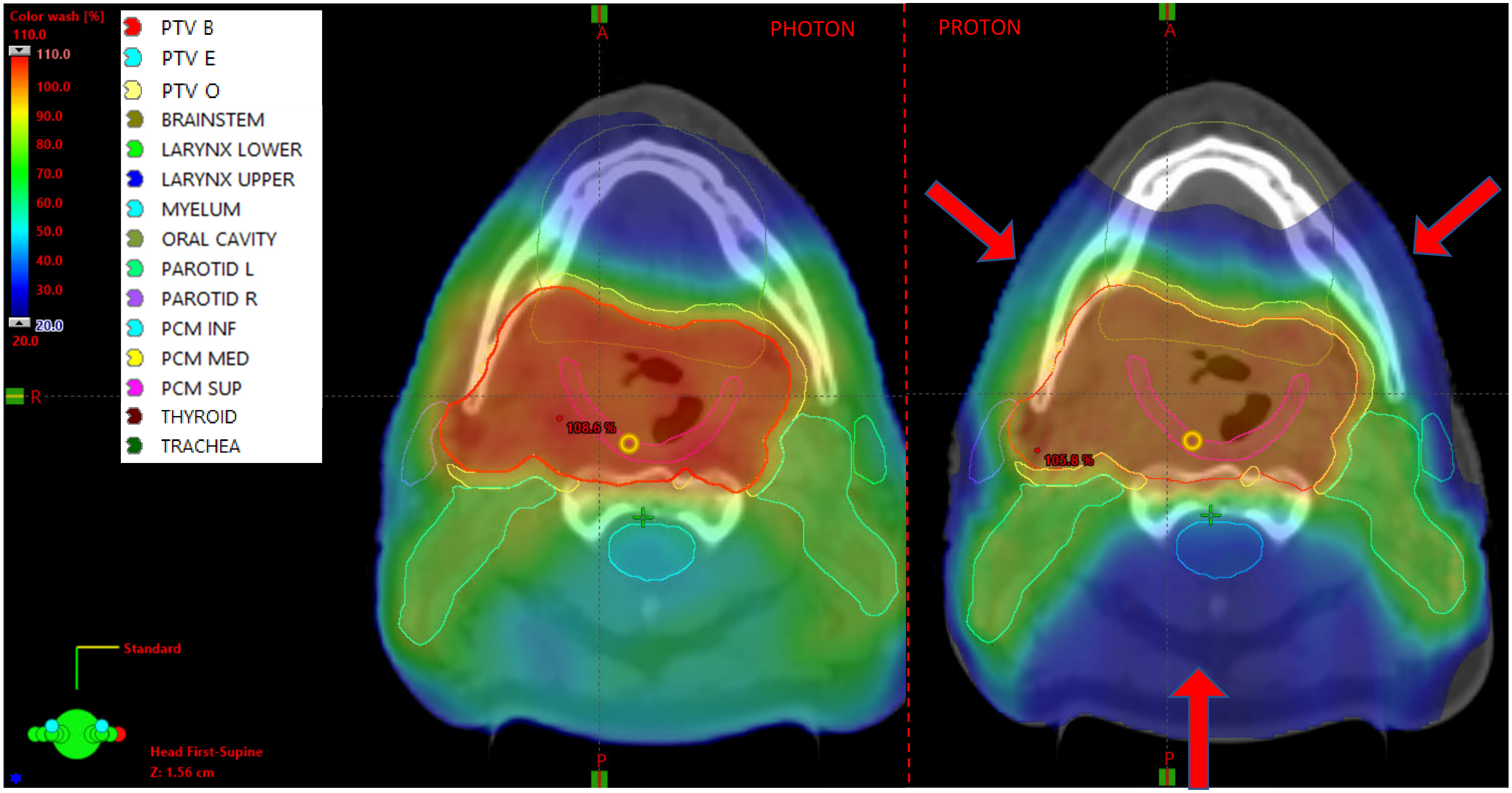

Supplementary Figure A1: Transversal slices of the auto-created photon (left) and proton (right) KBP dose distributions. The red arrows indicate the proton beam arrangement and the yellow circle marks the isocenter.

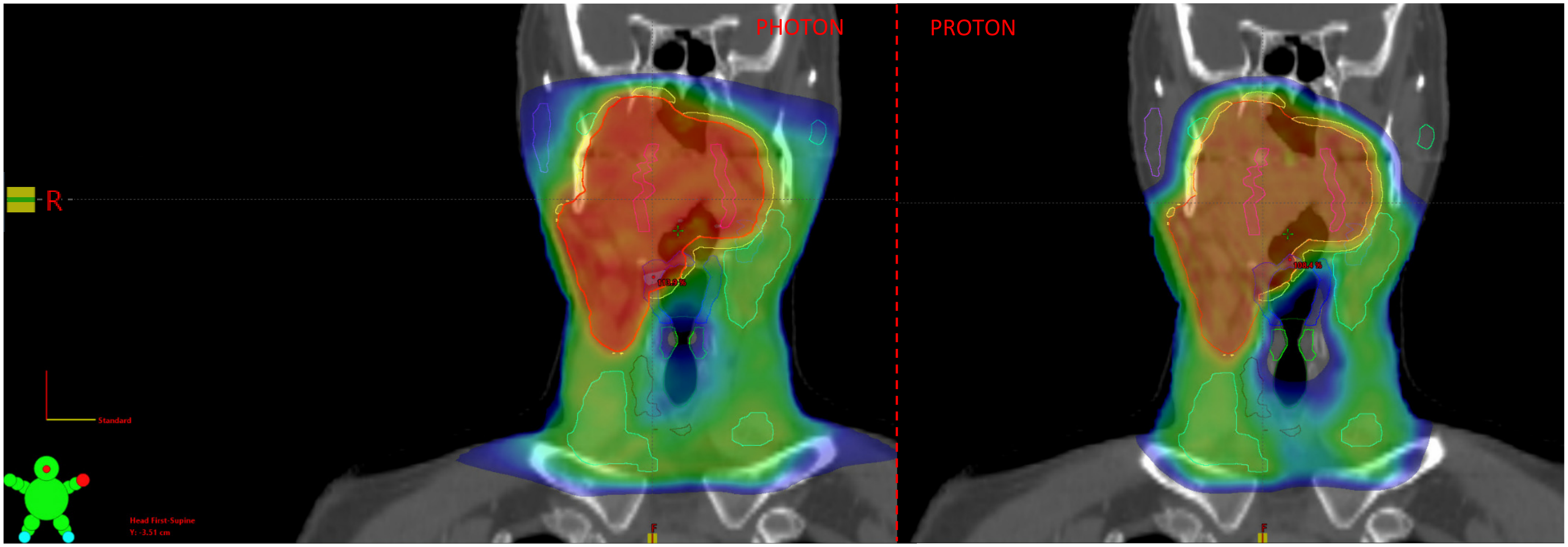

Supplementary Figure A2: Frontal slices of the auto-created photon (left) and proton (right) KBP dose distributions.

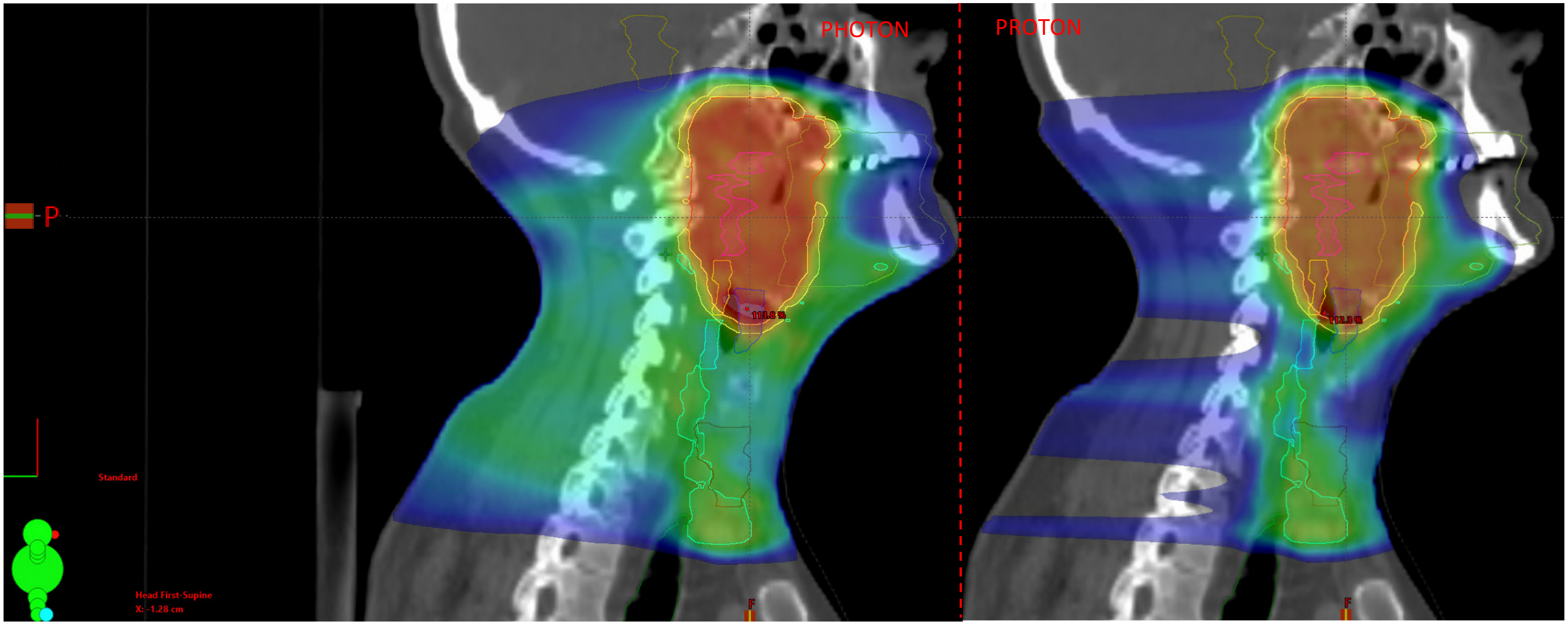

Supplementary Figure A3: Sagittal slices of the auto-created photon (left) and proton (right) KBP dose distributions.
