# Supplementary material for: Fast, Automated, Knowledge-Based Treatment Planning for Selecting Patients for Proton Therapy Based on Normal Tissue Complication Probabilities
Source: Adv Radiat Oncol. 2022 Jan 28;7(4):100903. doi: 10.1016/j.adro.2022.100903 (PMC8904224; doi:10.1016/j.adro.2022.100903)
Supplement: Supplementary file 2 [file mmc2.pdf]

Supplementary Table B1: Variable coefficients used to estimate the NTCP values[5].

VARIABLES	ENDPOINTS			
	Xerostomia after 6 months		Dysphagia after 6 months	
	Grade ≥ 2	Grade ≥ 3	Grade ≥ 2	Grade ≥ 3
Constant (β_0)	-2.2951	-3.7286	-4.0536	-7.6174
$\sqrt{\mathrm{D_{mean}}}(I.Parotid) + \sqrt{\mathrm{D_{mean}}}(C.Parotid)$	0.0996	0.0855		
$\mathrm{D_{mean}}(Both\ Submandibulars)$	0.0182	0.0156		
$\mathrm{D_{mean}}(Oral\ Cavity)$			0.0300	0.0259
$\mathrm{D_{mean}}(PCM\ Sup)$			0.0236	0.0203
$\mathrm{D_{mean}}(PCM\ Med)$			0.0095	0.0303
$\mathrm{D_{mean}}(PCM\ Inf)$			0.0133	0.0341
Primary Tumor Location in Pharynx			-0.6281	0.0387
