# Supplementary material for: Fast, Automated, Knowledge-Based Treatment Planning for Selecting Patients for Proton Therapy Based on Normal Tissue Complication Probabilities
Source: Adv Radiat Oncol. 2022 Jan 28;7(4):100903. doi: 10.1016/j.adro.2022.100903 (PMC8904224; doi:10.1016/j.adro.2022.100903)
Supplement: Supplementary file 3 [file mmc3.pdf]

Supplementary Material C: Application of RapidPlan prediction confidence boundaries for patient selection.

A major problem in using the RapidPlan dose predictions for patient selection was due to the selection pipeline consisting of a series of hard thresholds; if any one of the thresholds is exceeded due to erroneous prediction, the patient is erroneously indicated for protons. We explored the possibility to use the prediction confidence intervals to increase the accuracy so that a patient is indicated for one of the modalities only if both the upper and lower confidence boundaries (±1σ) do/do not cross the threshold. This approach is illustrated in Supplementary Figure S1, where patient 2 is indicated for photons due to the thresholds not being crossed by either boundary, patient 14 is indicated for protons due to the threshold for grade 3 dysphagia being crossed by both boundaries, and patient 22 is forwarded to KBP creation, as there are thresholds being crossed by one of the boundaries.

This approach indicates 6/14 patients directly for protons/photons, all in accordance with the KBP-indicated treatment modalities. This leaves 52 patients (72%) requiring KBPs to be created, which means that the gain of selection based on only the predictions with confidence intervals is small, and we recommend the actual automated plan making for all the patients in order to save time.

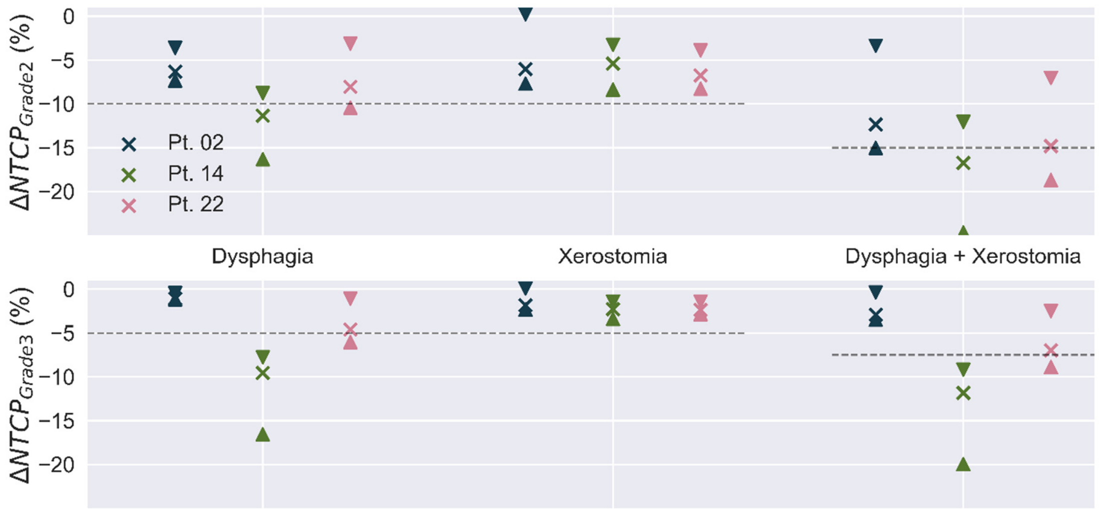

Supplementary Figure C1: Illustration of confidence intervals-based decision support with three patients using photon-KBP and RapidPlan predictions for protons (proton plan NTCP minus photon plan NTCP). The triangles and crosses indicate the predicted upper and lower bounds of the intervals and the achieved ΔNTCP values, respectively. The dotted lines indicate the patient selection thresholds.
